# Supplementary material for: Visualizing cortical laminar architecture in the living human brain using next-generation ultra-high-gradient diffusion MRI
Source: Commun Biol. 2026 Mar 23;9:651. doi: 10.1038/s42003-026-09887-2 (PMC13172339; doi:10.1038/s42003-026-09887-2)
Supplement: Supplementary file 1 — Supplementary Information [file 42003_2026_9887_MOESM1_ESM.pdf]

Visualizing cortical laminar architecture in the living human brain using next-generation ultra-high-gradient diffusion MRI

Hansol Lee[1,2,3,¶], Yixin Ma[1,2,¶], Kwok-Shing Chan[1,2], Eva A. Krijnen[4,5], Laleh Eskandarian[1,2], Aneri Bhatt[1,2], Julianna Gerold[1,2], Mirsad Mahmutovic[7], Oula Puonti[1,6], Xiangrui Zeng[1,2], Lucas Jacob Deden Binder[1,2], Bruce Fischl[1,2], Boris Keil[7,8,9], Gabriel Ramos-Llordén[1,2], Eric C. Klawiter[4], Hong-Hsi Lee[1,2,&,*], and Susie Y. Huang[1,2,&,*]

[1]Athinoula A. Martinos Center for Biomedical Imaging, Department of Radiology, Massachusetts General Hospital, Charlestown, MA, United States

[2]Harvard Medical School, Boston, MA, United States

[3]Department of Biomedical Engineering, Ulsan National Institute of Science and Technology, Ulsan, South Korea

[4]Department of Neurology, Massachusetts General Hospital, Harvard Medical School, Boston, MA, United States

[5]MS Center Amsterdam, Anatomy and Neurosciences, Amsterdam Neuroscience, Amsterdam UMC location VUmc, Amsterdam, The Netherlands

[6]Danish Research Centre for Magnetic Resonance, Centre for Functional and Diagnostic Imaging and Research, Copenhagen University Hospital - Amager and Hvidovre, Copenhagen, Denmark

[7]Institute of Medical Physics and Radiation Protection, TH-Mittelhessen University of Applied Sciences, Giessen, Germany

[8]LOEWE Research Cluster for Advanced Medical Physics in Imaging and Therapy (ADMIT), TH-Mittelhessen University of Applied Sciences, Giessen, Germany

[9]Department of Diagnostic and Interventional Radiology, University Hospital Marburg, Philipps University of Marburg, Marburg, Germany

[¶] Hansol Lee and Yixin Ma contributed equally to this work as first authors.

[&] Susie Y. Huang and Hong-Hsi Lee jointly supervised this work as corresponding authors.

* Corresponding authors:

Susie Y. Huang

E-mail : susie.huang@mgh.harvard.edu (SYH)

Hong-Hsi Lee

E-mail : hlee84@mgh.harvard.edu (HHL)

Supplementary Note 1

Self-similarity-based super-resolution imaging processing

Methods

A self-similarity-based super-resolution technique was applied to enhance the spatial resolution of dMRI data (2 mm isotropic), reducing partial volume effects and enhancing anatomical detail[1-3]. This method incorporates high spatial frequency information from high-resolution (1 mm isotropic) T_1-weighted images to guide the upsampling of low-resolution diffusion-weighted images (DWIs) (**Supplementary Fig. S1**). The algorithm defines a local 5×5×5 search window around each voxel. For a center voxel and a surrounding voxel within this window, interpolation weights are computed based on the intensity differences (1) between the 3×3×3 voxel patches centered at the two voxels in the DWI and (2) between the two voxels in the co-registered T_1-weighted image. Voxel distance is not considered in the weighting, enabling similarity-based interpolation driven purely by local signal characteristics. The T_1-weighted image was co-registered to the up-sampled b0 image and DWI space using FreeSurfer "bbregister" function and served as the anatomical reference. Initial up-sampling of the b0 image and DWI to 1 mm isotropic resolution was performed using nearest-neighbor interpolation via the "mrgrid" function in MRtrix3. To progressively refine patch matching, a hyperparameter h was increased over six iterations (e.g., 1, 2, 4, 6, 8, 16). Data fidelity was maintained throughout the process by enforcing a consistency constraint: after each iteration, the down-sampled version of the high-resolution estimates was required to match the original low-resolution DWI. This iterative correction ensured preservation of diffusion contrast and stable convergence. The super-resolution algorithm was implemented in-house as the "mrsupres_voxel" function (https://github.com/yixinma9/super_resolution), fully compatible with the MRtrix3 framework. To support efficient processing of large-scale datasets, key computational steps, including patch-based similarity computation, image updating, and data consistency enforcement, were parallelized using multi-threading.

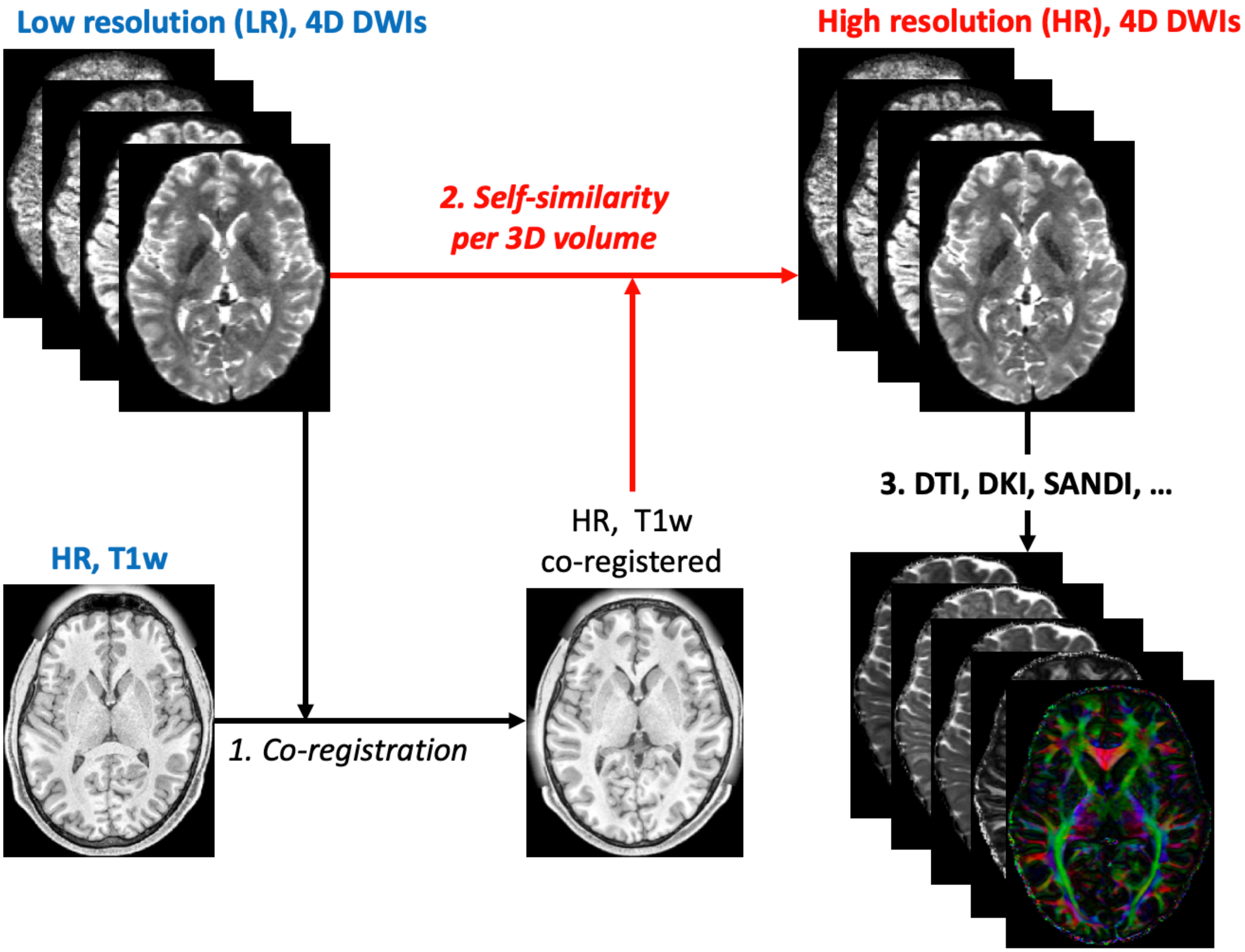

Supplementary Figure S1. Overview of the self-similarity-based super-resolution pipeline for diffusion MRI. Step 1: Low-resolution (LR) 4D diffusion-weighted images (DWIs) and b0 images were linearly interpolated to increase the matrix size by a factor of two. Then, a high-resolution (HR) T_1-weighted anatomical image was co-registered to the interpolated b0 images. **Step 2:** The co-registered T_1-weighted image provided high spatial frequency information to guide patch-based self-similarity super-resolution technique applied to each 3D DWI volume. **Step 3:** The super-resolution algorithm iteratively reconstructs high-resolution (HR) DWIs by transferring structural detail from the anatomical image and local signal similarities in the diffusion data, yielding sharper contrasts and reduced partial volume effects. After that, we can fit any biophysical model to the HR 4D DWIs.

Results

The high-resolution (1 mm isotropic) dMRI-derived SANDI metrics generated using the super-resolution technique improved the visualization of detailed microstructural features and effectively reduced partial volume effects compared to the lower-resolution data (**Supplementary Fig. S2**).

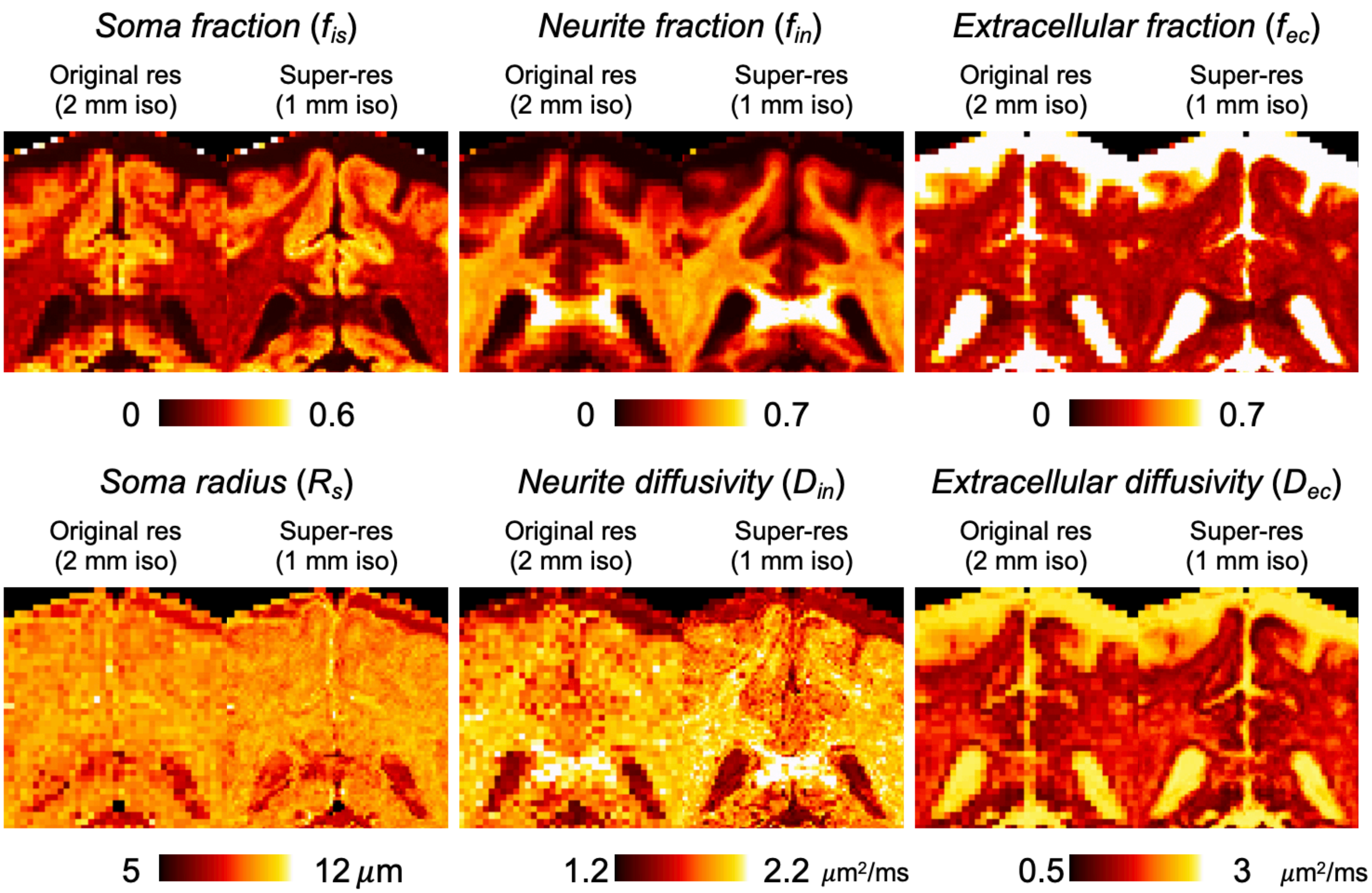

Supplementary Figure S2. SANDI-derived microstructural metrics maps at original resolution (2 mm isotropic) and super-resolution (1 mm isotropic). The super-resolution images are derived from self-similarity-based super-resolution image processing.

When comparing super-resolution with conventional interpolation methods, super-resolution produced images with improved fine cortical features and sharper gray-white matter and cortex-CSF contrast relative to nearest-neighbor or cubic interpolation (**Supplementary Fig. S3a**). Quantitative evaluation using down-sampled data further confirmed its superior reconstruction performance, with super-resolution achieving the lowest mean squared error (MSE) and mean absolute error (MAE) values and the highest structural similarity index measure (SSIM) when compared to the ground-truth 2 mm image (**Supplementary Fig. S3b**).

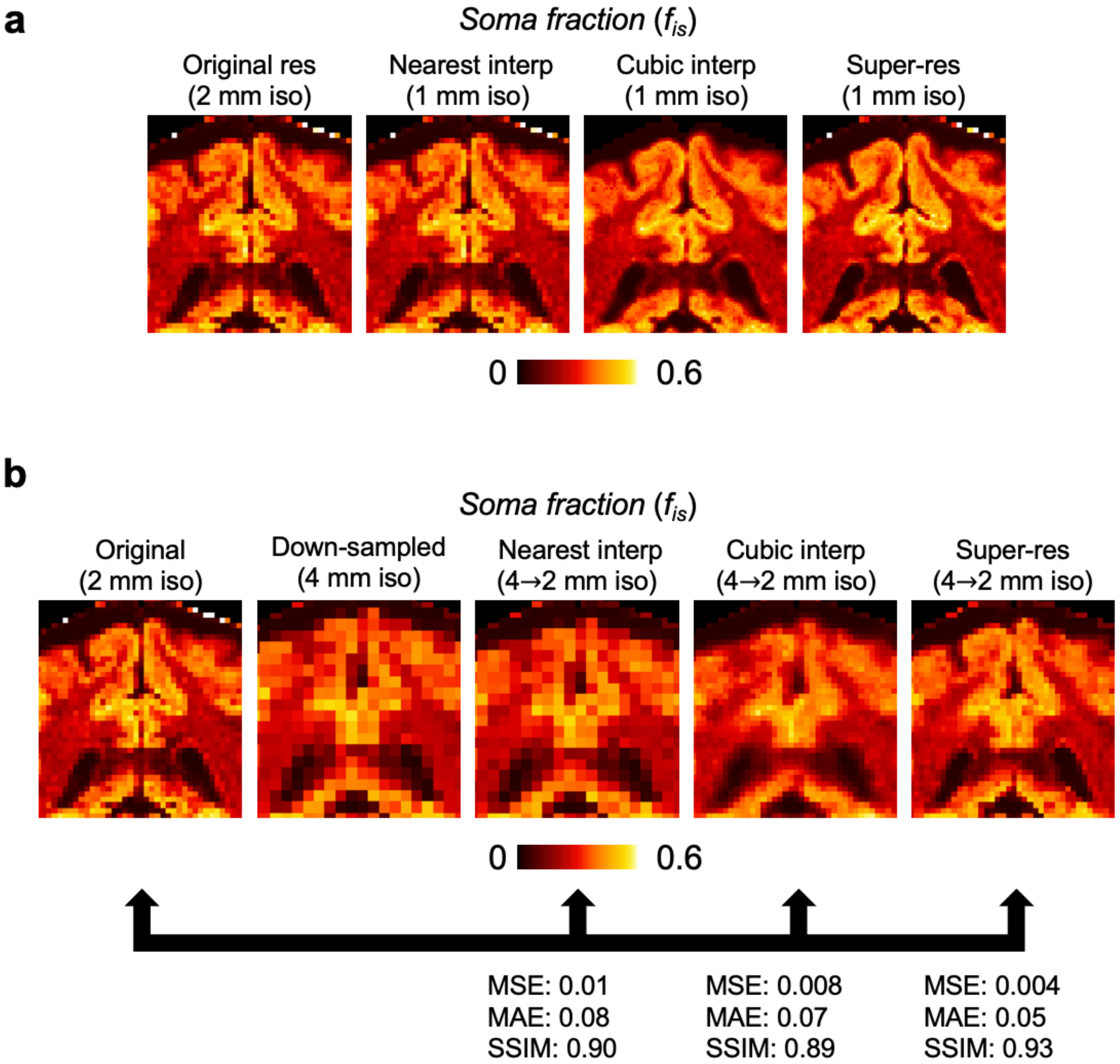

Supplementary Fig. S3. Super-resolution image processing for SANDI-derived intra-soma signal fraction f_{is} maps. a Intra-soma signal fraction f_{is} at 2 mm isotropic resolution and the corresponding 1 mm isotropic images obtained using different upsampling methods (nearest-neighbor interpolation, cubic interpolation, and super-resolution). **b** Quantitative validation comparing the ground-truth 2 mm image with a 4 mm down-sampled version that was subsequently upsampled back to 2 mm using the same methods.

Discussion

This study demonstrated the substantial advantages of applying a self-similarity-based super-resolution technique to dMRI data, enhancing the capabilities of biophysical dMRI modeling by leveraging the high-resolution details from T_1-weighted anatomical images. By upsampling the lower resolution dMRI data, we

effectively generated high-resolution SANDI metrics that revealed finer structural details and substantially reduced partial volume effects. Compared to traditional interpolation methods like trilinear, B-spline, and cubic approaches, this technique significantly improves SNR and minimizes artifacts near tissue boundaries [2]. In the maps of the intra-soma signal fraction f_{is}, this improvement was particularly noticeable in complex structural regions and along brain boundaries, where tissue contacts cerebrospinal fluid. Future research integrating sophisticated image processing techniques with high-resolution dMRI acquisitions, leveraging the ultra-high-performance gradient system of Connectome 2.0 to achieve sub-millimeter resolution, holds the potential to advance microstructural analyses and detailed characterization of cellular architecture.

Supplementary Note 2

Noise propagation analysis of the SANDI model fitting using the Connectome 2.0 protocol with varying soma diffusivity D_{is}

Methods

The randomly distributed parameters were simulated for the SANDI fitting with an SNR of 50 in the presence of Rician noise. A total of 1500 signals were generated from the randomly distributed parameters: intra-soma signal fraction f_{is} between 0.1 and 0.5, intra-neurite signal fraction f_{in} between 0.1 and 0.5, extracellular signal fraction $f_{ec} = 1 - f_{is} - f_{in}$, soma radius R_s between 5 and 12 µm, intra-neurite diffusivity D_{in} between 1 and 3 µm^2/ms, and extracellular diffusivity D_{ec} between 1 and 3 µm^2/ms, with the three different intrinsic soma diffusivity D_{is} values of 1, 2, and 3 µm^2/ms. These simulated signals were then fitted using the SANDI model for the Connectome 2.0 (δ = 6 ms and Δ = 13 ms) protocol, incorporating two intrinsic soma diffusivity D_{is} values, 2 µm^2/ms and 3 µm^2/ms, to evaluate the robustness of the parameter estimation considering variations in intrinsic soma diffusivity D_{is} values. The accuracy and precision of the model fitting were assessed by comparing the fitted parameters with their ground-truth values.

Results

The SANDI model fitting reliably estimated microstructural parameters, specifically f_{is}, f_{in}, f_{ec}, and D_{ec}, with high accuracy and precision under varying parameter distributions and soma diffusivity D_{is} used for diffusion signal generation and SANDI fitting (**Supplementary Figure S4**). The intra-soma signal fraction f_{is} was overestimated for lower ground truth values when the intrinsic soma diffusivity D_{is} used for signal generation was 1 or 2 µm^2/ms, and the intrinsic soma diffusivity D_{is} used for SANDI fitting was 3 µm^2/ms (indicated by red arrows). Improved estimation of intra-soma signal fraction f_{is} and extracellular signal fraction f_{ec} was observed when the intrinsic soma diffusivity D_{is} used for SANDI fitting was 2 µm^2/ms. The precision of the intra-neurite signal fraction f_{in} was the highest among the fitted parameters. The fitted soma radius R_s showed a slight positive

correlation with the ground truth values; however, it was consistently overestimated for smaller radii and underestimated for larger radii exceeding 9 μm. The fitted intra-neurite diffusivity D_{in} showed no apparent variation across the range of ground truth values.

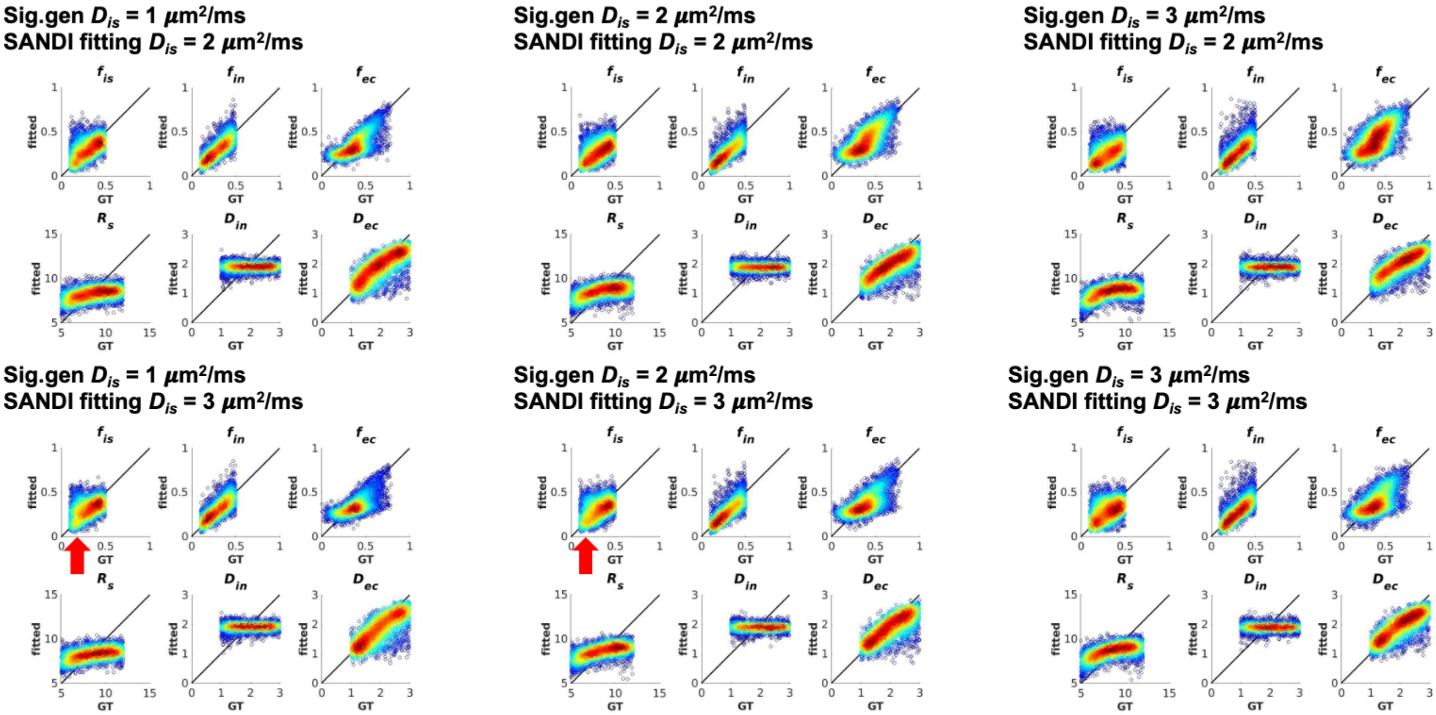

Supplementary Figure S4. Noise propagation analysis in the SANDI model fitting with varying intrinsic soma diffusivity D_{is}. GT denotes ground truth values, while fitted values are derived from the SANDI fitting process. Red arrows indicate overestimation of intra-soma signal fraction f_{is} at low ground truth values when lower intrinsic soma diffusivity D_{is} was used for signal generation, but higher intrinsic soma diffusivity D_{is} for model fitting.

Discussion

The noise propagation analysis of the SANDI model fitting demonstrated robust performance under varying parameter distributions for Connectome 2.0 protocols, effectively estimating key microstructural parameters such as the intra-soma signal fraction f_{is}, the intra-neurite signal fraction f_{in}, the extracellular signal fraction f_{ec}, and extracellular diffusivity D_{ec}. The model's reliability was evident under different assumptions for intrinsic soma diffusivity D_{is}. Our analysis revealed that using intrinsic soma diffusivity D_{is} = 3 μm^2/ms in the fitting process led to an overestimation of intra-soma signal fraction f_{is}, particularly when the true intrinsic soma diffusivity D_{is} values were lower (1 or 2 μm^2/ms). In contrast, fixing intrinsic soma diffusivity D_{is} at 2 μm^2/ms yielded improved estimation accuracy for intra-soma signal fraction f_{is} and extracellular signal fraction f_{ec}. These

results emphasize the sensitivity of the SANDI model to various intrinsic soma diffusivity D_{is} values. The choice of intrinsic soma diffusivity D_{is} = 2 μm^2/ms represents an optimal compromise within the biological range of 1-3 μm^2/ms, resulting in more reliable parameter estimation across varying tissue conditions. This choice is further supported by previous studies, including Ianuş, et al., 2022, which used D_{is} = 2 μm^2/ms in mouse *in vivo* applications of the SANDI model[4]. Additionally, while the model showed the highest precision in estimating f_{in} among all parameters, the estimation of soma radius R_s exhibited systematic biases, overestimating smaller radii and underestimating those exceeding 9 μm, suggesting potential limitations in radius estimation that warrant careful interpretation.

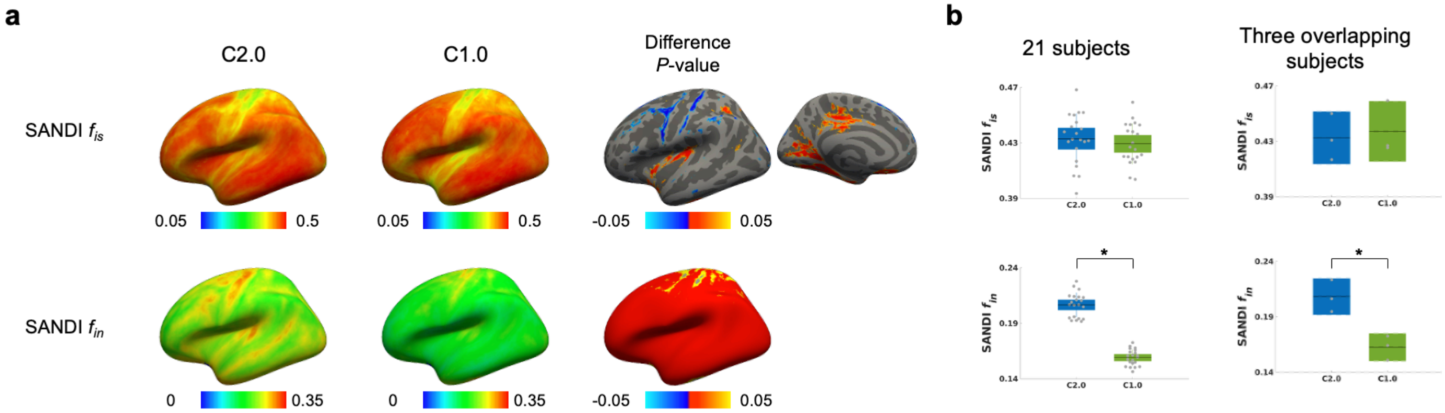

Supplementary Figure S5. SANDI-derived microstructural metrics on Connectome 2.0 (C2.0) and Connectome 1.0 (C1.0) scanners. a Cortical maps of the intra-soma signal fraction f_{is} and intra-neurite signal fraction f_{in} derived from SANDI, averaged across 21 individuals. The third column shows the *P*-values of vertex-wise differences in these metrics between Connectome 2.0 and Connectome 1.0, indicating regions with statistically significant differences. **b** Boxplots summarizing intra-soma signal fraction f_{is} and intra-neurite signal fraction f_{in} across individuals (N=21 for both scanners), including a subset of participants who were scanned on both scanners (N=3), with statistically significant differences of f_{in} (∗: FDR-P<0.05) between Connectome 2.0 and Connectome 1.0 scanners. The box represents the 95% confidence interval (mean ± 1.96 standard error of the mean). The solid horizontal line indicates the mean, and the thinner vertical lines denote ± 1 standard deviation. Individual dots correspond to individual subjects.

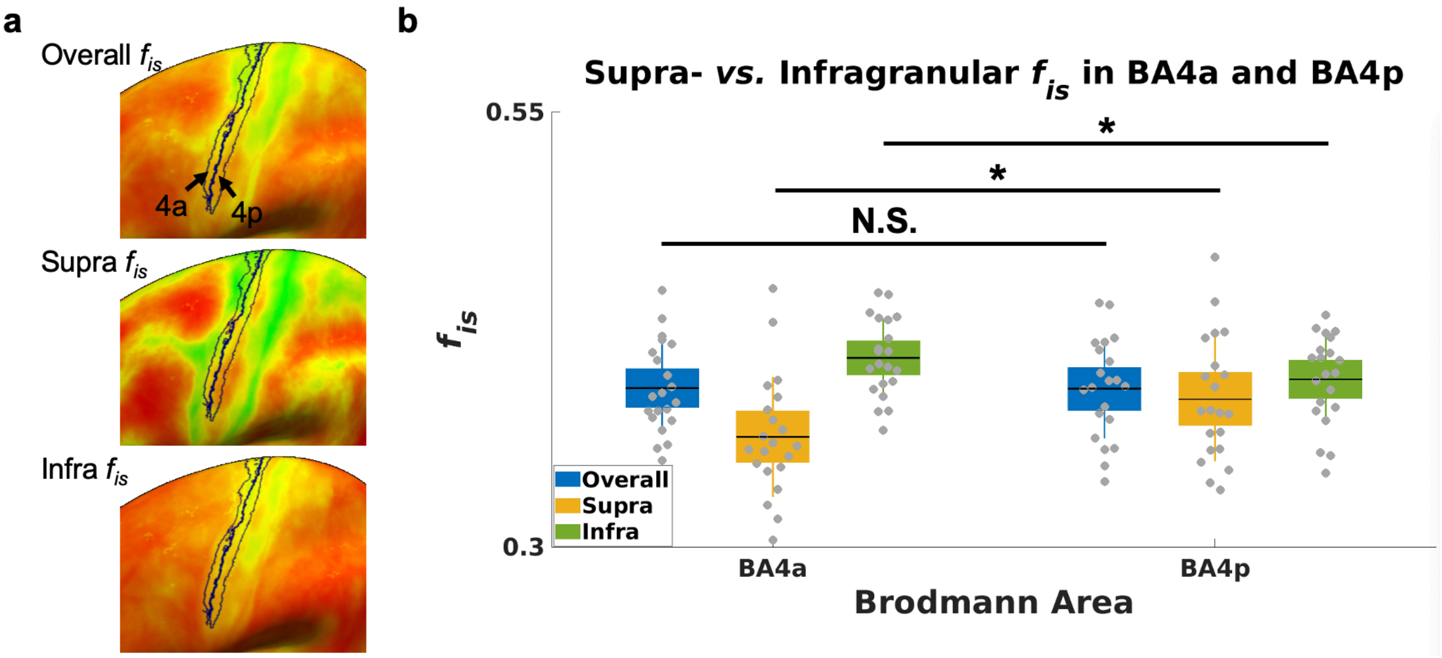

Supplementary Figure S6. Laminar-specific intra-soma signal fraction f_{is} distinguishing adjacent motor subregions BA4a and BA4p. a Surface maps of overall, supragranular, and infragranular intra-soma signal fraction f_{is}; black contours outlining BA4a and BA4p boundaries. **b** Boxplots of intra-soma signal fraction f_{is} across overall, supragranular, and infragranular layers for each Brodmann area, with statistically significant differences (∗: FDR-P<0.05). The box represents the 95% confidence interval (mean ± 1.96 standard error of the mean). The solid horizontal line indicates the mean, and the thinner vertical lines denote ± 1 standard deviation. Individual dots correspond to individual subjects.

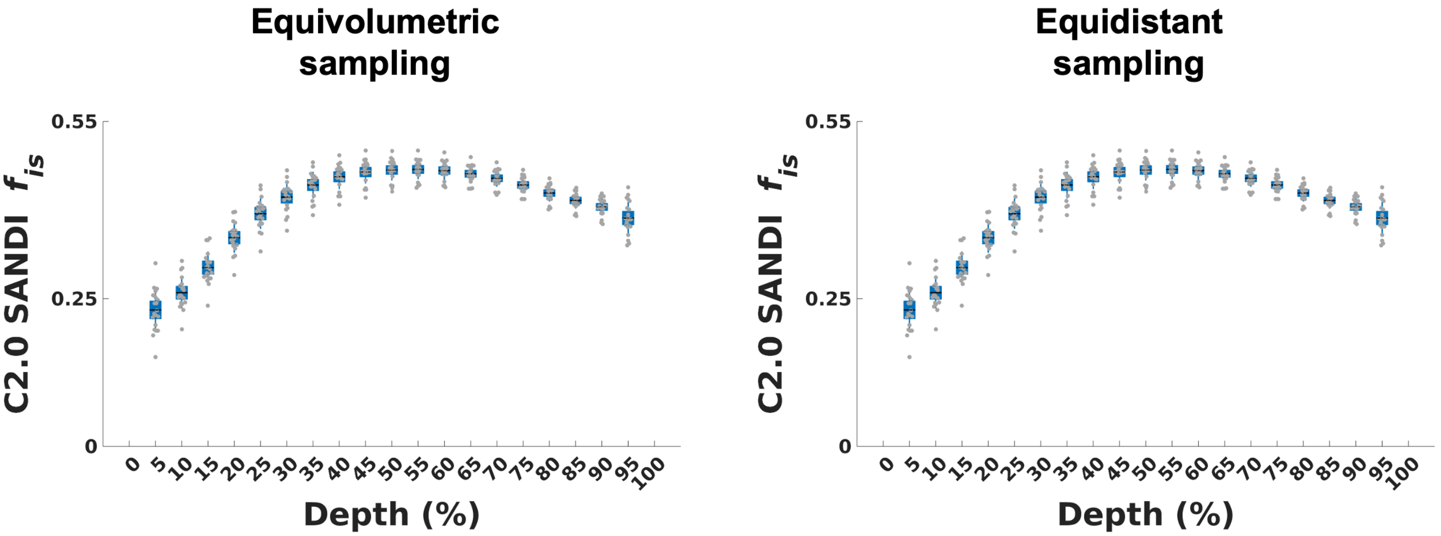

Supplementary Figure S7. Depth-dependent intra-soma signal fraction f_{is} profiles across the whole cortex, derived using equivolumetric sampling and equidistant sampling implemented in LayNii toolbox[5]. The box represents the 95% confidence interval (mean ± 1.96 standard error of the mean). The solid horizontal line indicates the mean, and the thinner vertical lines denote ± 1 standard deviation. Individual dots correspond to individual subjects.

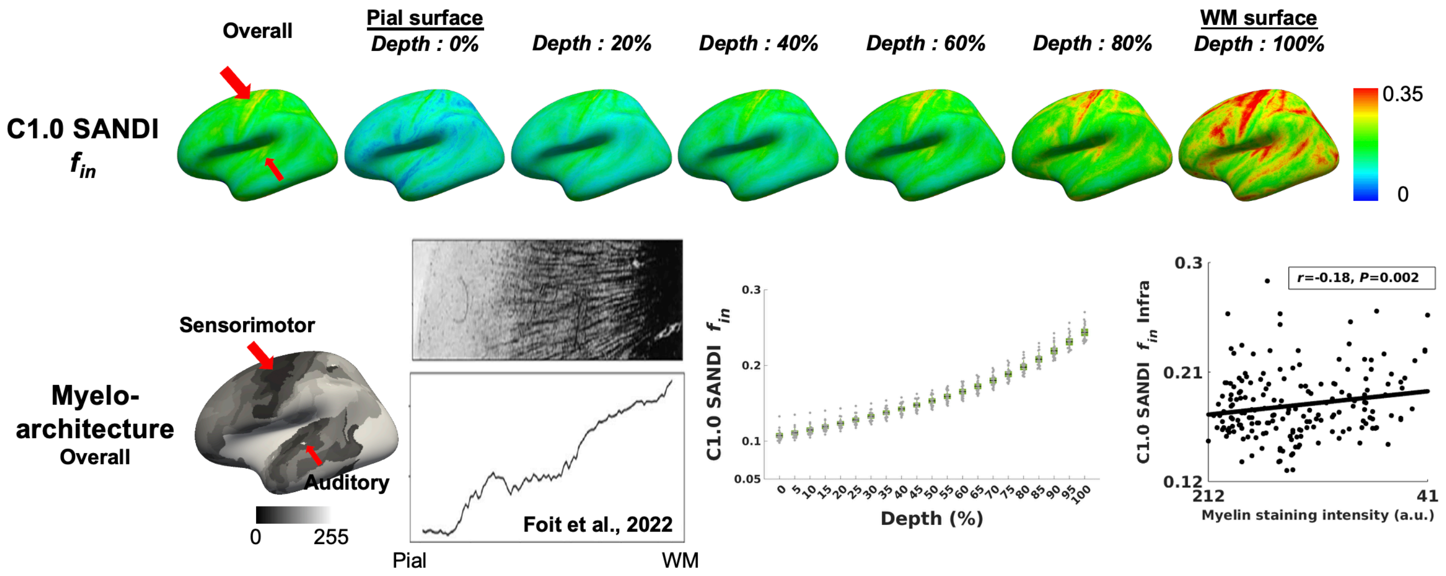

Supplementary Figure S8. SANDI-derived intra-neurite signal fraction f_{in} on the Connectome 1.0 scanner, alongside the myelin staining data from the myeloarchitecture atlas across the cortical depths. In the myelin staining data, darker colors (i.e., lower intensity values) correspond to higher myelin concentration across regions defined by Nieuwenhuys' parcellation. The data of myelin staining intensity across cortical depths and the figure of histology are sourced from Foit et al., *NeuroImage* (2022)[6], with permission from Elsevier. The box represents the 95% confidence interval (mean ± 1.96 standard error of the mean). The solid horizontal line indicates the mean, and the thinner vertical lines denote ± 1 standard deviation. Individual dots correspond to individual subjects.

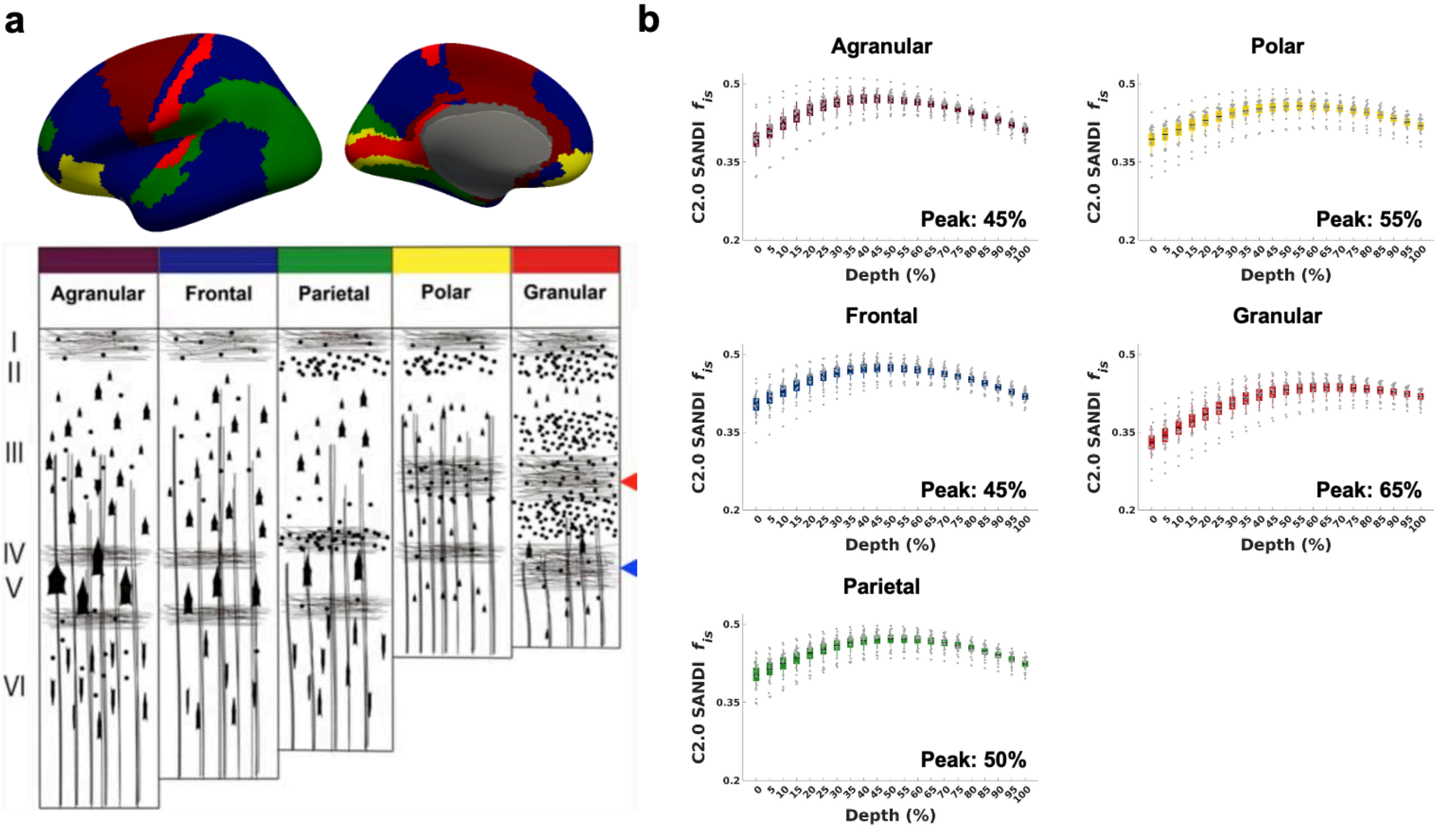

Supplementary Figure S9. Depth-dependent SANDI-derived intra-soma signal fraction f_{is} across von Economo cortical types. a von Economo's cytoarchitectonic cortical type parcellation displayed on the fsaverage cortical surface, alongside schematic laminar cytoarchitecture for each cortex type adapted from Fukutomi et al., *NeuroImage* (2019)[7]. **b** Depth-dependent profiles of the SANDI-derived intra-soma signal fraction f_{is} and BigBrain Merker staining intensity for each cortical type. The box represents the 95% confidence interval (mean ± 1.96 standard error of the mean). The solid horizontal line indicates the mean, and the thinner vertical lines denote ± 1 standard deviation. Individual dots correspond to individual subjects.

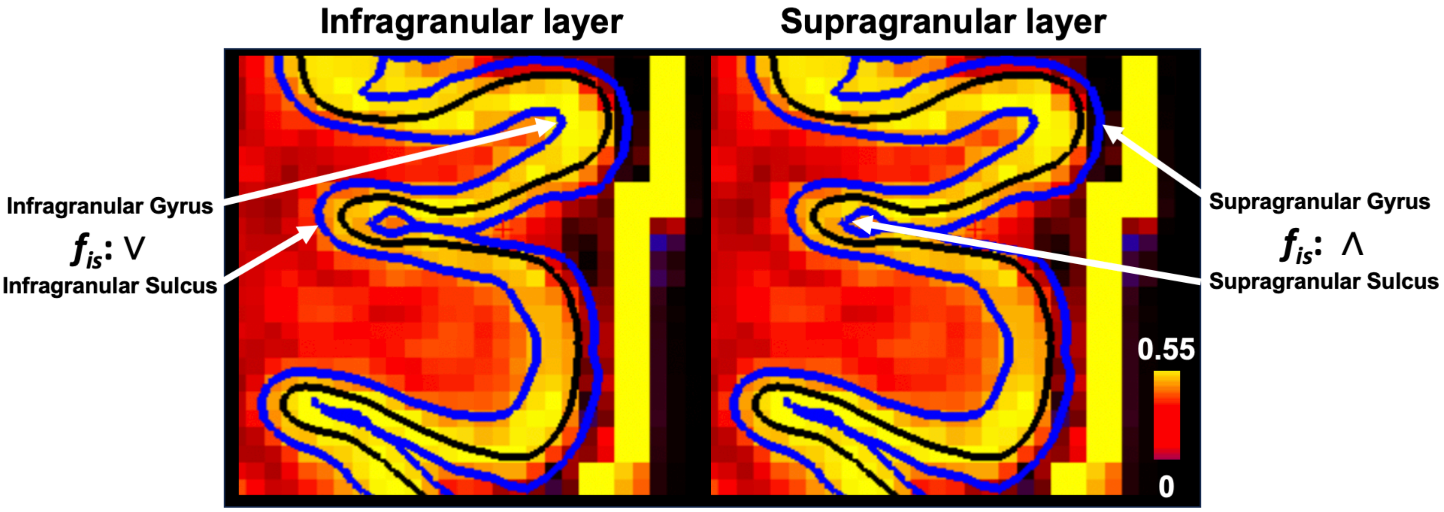

Supplementary Figure S10. Representative intra-soma signal fraction f_{is} maps illustrating gyral and sulcal microstructural differences across cortical layers. f_{is} maps in a representative gyral/sulcal pair show the microstructural differences between gyral and sulcal regions within the infragranular layer and the supragranular layer. These maps highlight the opposing gyral-sulcal relationships observed across cortical depths, with higher f_{is} values in the infragranular gyrus (compared with infragranular sulcus) and higher f_{is} values in the supragranular sulcus (compared with supragranular gyrus). Blue contours denote white-gray matter boundary (inner contour) and pial surface (outer contour), respectively, and the black contour indicates the boundary separating the supragranular and infragranular layers.

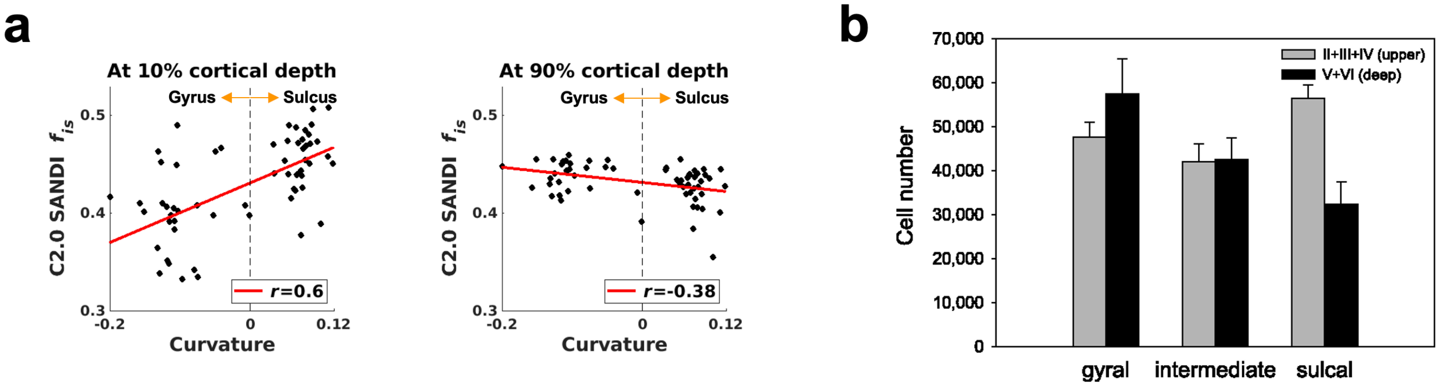

Supplementary Figure S11. Curvature-dependent intra-soma signal fraction f_{is} and histological cell density distribution. a Scatter plots show the relationships between curvature and f_{is} at 10% and 90% cortical depths. **b** Histologically derived cell number profiles for different cortical folding patterns adapted from Hilgetag et al., *PLoS Computational Biology* (2006)[8] show corresponding layer-specific trends.

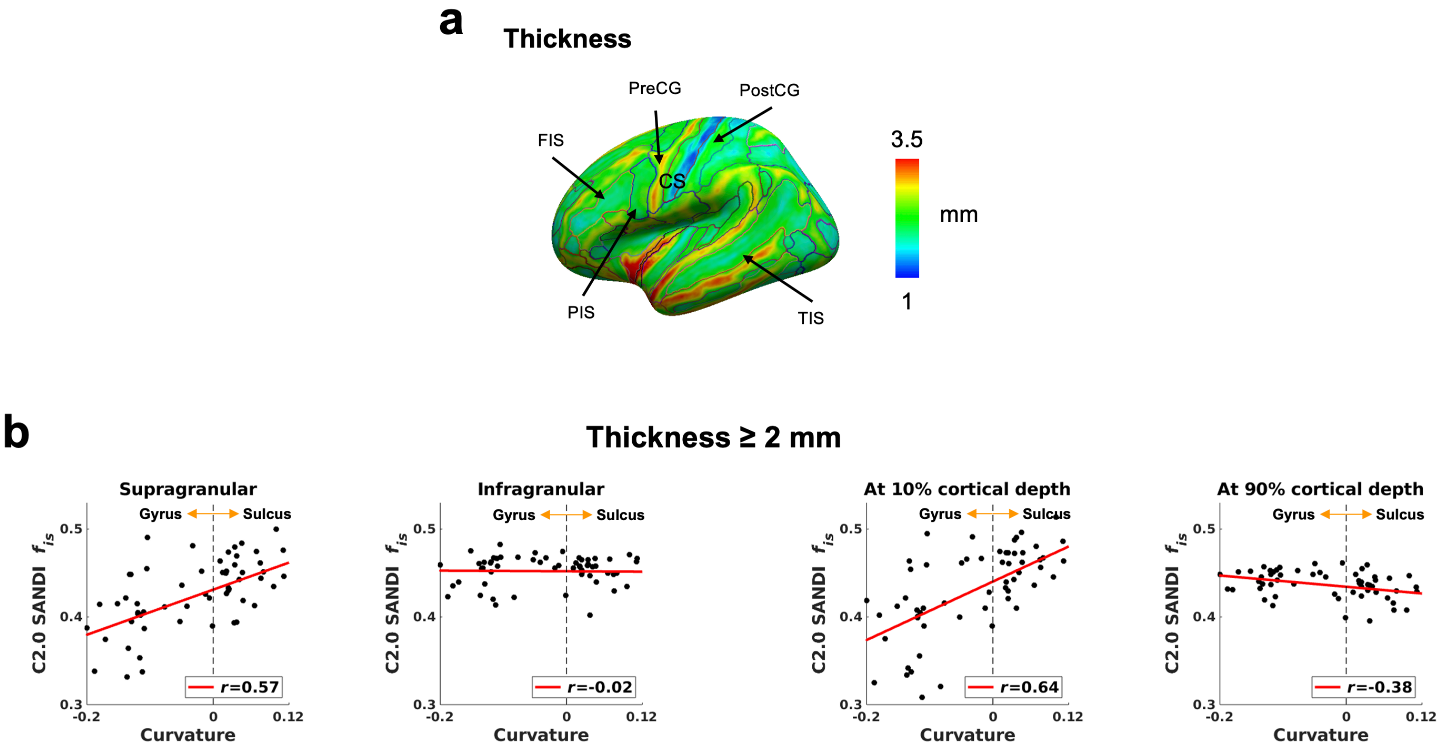

Supplementary Figure S12. Relationship between intra-soma signal fraction f_{is} and cortical curvature with thickness ≥ 2 mm. a Cortical thickness map displayed on the fsaverage surface, with labels from the aparc.a2009s.annot parcellation. **b** Scatter plots show the relationships between curvature and f_{is} in supragranular and infragranular layers of cortical regions with thickness ≥ 2 mm, as well as at 10% and 90% cortical depths. CS: central sulcus; FIS: frontal inferior sulcus; PIS: parietal inferior sulcus; PostCG: postcentral gyrus; PreCG: precentral gyrus; TIS: temporal inferior sulcus.

References

1 Manjón, J. V., Coupé, P., Buades, A., Collins, D. L. & Robles, M. MRI superresolution using self-similarity and image priors. *International journal of biomedical imaging* **2010**, 425891 (2010).

2 Coupé, P., Manjón, J. V., Chamberland, M., Descoteaux, M. & Hiba, B. Collaborative patch-based super-resolution for diffusion-weighted images. *NeuroImage* **83**, 245-261 (2013).

3 Lee, H. H. *et al.* SUper-REsolution TRACTography (SURE-TRACT) pipeline using self-similarity between diffusional and anatomical images. *Proc of ISMRM. Montreal* **27**, 167 (2019).

4 Ianus, A. *et al.* Soma and Neurite Density MRI (SANDI) of the in-vivo mouse brain and comparison with the Allen Brain Atlas. *Neuroimage* **254**, 119135 (2022). https://doi.org:10.1016/j.neuroimage.2022.119135

5 Huber, L. R. *et al.* LayNii: A software suite for layer-fMRI. *NeuroImage* **237**, 118091 (2021).

6 Foit, N. A. *et al.* A whole-brain 3D myeloarchitectonic atlas: Mapping the Vogt-Vogt legacy to the cortical surface. *Neuroimage* **263**, 119617 (2022).

7 Fukutomi, H. *et al.* Neurite imaging reveals microstructural variations in human cerebral cortical gray matter. *Neuroimage* **182**, 488-499 (2018).

8 Hilgetag, C. C. & Barbas, H. Role of mechanical factors in the morphology of the primate cerebral cortex. *PLoS Comput Biol* **2**, e22 (2006). https://doi.org:10.1371/journal.pcbi.0020022
