# Supplementary material for: Visualizing cortical laminar architecture in the living human brain using next-generation ultra-high-gradient diffusion MRI
Source: Commun Biol. 2026 Mar 23;9:651. doi: 10.1038/s42003-026-09887-2 (PMC13172339; doi:10.1038/s42003-026-09887-2)
Supplement: Supplementary file 2 — Description of Additional Supplementary Materials [file 42003_2026_9887_MOESM2_ESM.pdf]

Description of Additional Supplementary Files

File name: Supplementary Data 1

Description: Numerical source data for figures presented in the manuscript
