# Supplementary material for: Visualizing cortical laminar architecture in the living human brain using next-generation ultra-high-gradient diffusion MRI
Source: Commun Biol. 2026 Mar 23;9:651. doi: 10.1038/s42003-026-09887-2 (PMC13172339; doi:10.1038/s42003-026-09887-2)
Supplement: Supplementary file 4 — Reporting Summary [file 42003_2026_9887_MOESM4_ESM.pdf]

nature portfolio

Corresponding author(s): Susie Y. Huang
Hong-Hsi Lee

Last updated by author(s): Feb 28, 2026

Reporting Summary

Nature Portfolio wishes to improve the reproducibility of the work that we publish. This form provides structure for consistency and transparency in reporting. For further information on Nature Portfolio policies, see our Editorial Policies and the Editorial Policy Checklist.

Statistics

For all statistical analyses, confirm that the following items are present in the figure legend, table legend, main text, or Methods section.

n/a	Confirmed	
☐	☒	The exact sample size (*n*) for each experimental group/condition, given as a discrete number and unit of measurement
☐	☒	A statement on whether measurements were taken from distinct samples or whether the same sample was measured repeatedly
☐	☒	The statistical test(s) used AND whether they are one- or two-sided<br>*Only common tests should be described solely by name; describe more complex techniques in the Methods section.*
☐	☒	A description of all covariates tested
☐	☒	A description of any assumptions or corrections, such as tests of normality and adjustment for multiple comparisons
☐	☒	A full description of the statistical parameters including central tendency (e.g. means) or other basic estimates (e.g. regression coefficient) AND variation (e.g. standard deviation) or associated estimates of uncertainty (e.g. confidence intervals)
☐	☒	For null hypothesis testing, the test statistic (e.g. *F*, *t*, *r*) with confidence intervals, effect sizes, degrees of freedom and *P* value noted<br>*Give P values as exact values whenever suitable.*
☒	☐	For Bayesian analysis, information on the choice of priors and Markov chain Monte Carlo settings
☒	☐	For hierarchical and complex designs, identification of the appropriate level for tests and full reporting of outcomes
☐	☒	Estimates of effect sizes (e.g. Cohen's *d*, Pearson's *r*), indicating how they were calculated

Our web collection on statistics for biologists contains articles on many of the points above.

Software and code

Policy information about availability of computer code

Data collection

All imaging data were acquired using the Siemens Connectome 2.0 & Connectome 1.0 MRI scanners with a customized high-gradient diffusion protocol. The 3T Connectome 2.0 MRI scanner (MAGNETOM Connectom.X, Siemens Healthineers, Erlangen, Germany) is equipped with a Gmax of 500 mT/m and an SRmax of 600 T/m/s, using a custom-built 72-channel in vivo head coil for signal reception. The 3T Connectome 1.0 MRI scanner (MAGNETOM Connectom, Siemens Healthcare) was equipped with a Gmax of 300 mT/m and SRmax of 200 T/m/s using a custom-built 64-channel in vivo head coil.

Data analysis

dMRI data were preprocessed using an in-house script based on the DESIGNER pipeline. Raw dMRI data were corrected for Gibbs ringing artifact using the “mrdegibbs” function in MRtrix3, susceptibility and eddy current-induced distortions using the “topup” and “eddy” functions in FSL (https://fsl.fmrib.ox.ac.uk), followed by a gradient non-linearity correction.
The SANDI model was fitted to multi-shell dMRI signals averaged over gradient directions (spherical mean), employing the SANDI MATLAB toolbox (https://github.com/palombom/SANDI-Matlab-Toolbox-v1.0).
We processed 3D T1-weighted anatomical images using FreeSurfer (version 7.1.4, https://surfer.nmr.mgh.harvard.edu) through the standard “recon-all” pipeline for skull stripping, cortical gray matter parcellation, and cortical surface reconstruction.
The averaged non-diffusion-weighted image was aligned with the T1-weighted anatomical image using the “bbregister” function in FreeSurfer, which employs a boundary-based rigid body transformation with 6 degrees of freedom.
We then utilized the FreeSurfer commands “mri_compute_layer_fractions” and “mri_compute_layer_intensities” to extract SANDI-derived metrics across the whole cortical thickness as well as within supragranular and infragranular layers.
The cortex was further divided into 21 evenly spaced depth intervals (5% intervals), ranging from the pial surface (0% depth) to the white matter boundary (100% depth), for a depth-dependent analysis of microstructural features. SANDI metrics were extracted at these depths using the “mri_vol2surf” function in FreeSurfer based on the transformation information from the co-registration between the non-diffusion-

weighted image and the T1-weighted image. Individual subject data were aligned to the FreeSurfer "fsaverage" cortical surface template using the FreeSurfer "mri_vol2surf" function.

For manuscripts utilizing custom algorithms or software that are central to the research but not yet described in published literature, software must be made available to editors and reviewers. We strongly encourage code deposition in a community repository (e.g. GitHub). See the Nature Portfolio guidelines for submitting code & software for further information.

Data

Policy information about availability of data

All manuscripts must include a data availability statement. This statement should provide the following information, where applicable:

- Accession codes, unique identifiers, or web links for publicly available datasets
- A description of any restrictions on data availability
- For clinical datasets or third party data, please ensure that the statement adheres to our policy

A Connectome 2.0 diffusion MRI dataset (raw and preprocessed DWIs) is publicly available on OpenNeuro (https://doi.org/10.18112/openneuro.ds006181.v1.0.0). Connectome 1.0 DWI datasets are publicly available via Figshare (https://doi.org/10.6084/m9.figshare.c.5315474). All other data supporting the findings of this study are available from the corresponding author upon reasonable request, subject to applicable data-sharing agreements and conditions of reuse.

Research involving human participants, their data, or biological material

Policy information about studies with human participants or human data. See also policy information about sex, gender (identity/presentation), and sexual orientation and race, ethnicity and racism.

Reporting on sex and gender	Participants were divided into two groups: 21 individuals (14 females, 7 males; mean age: 29.0±4.5 years; age range: 19-37) underwent MRI scans using the newly installed 3T Connectome 2.0 MRI scanner (Gmax of 500 mT/m and maximum slew rate of 600 T/m/s). Age- and sex-matched 21 participants (14 females, 7 males; mean age: 28.7±6.2 years; age range: 19-40) were scanned on the 3T Connectome 1.0 scanner (Gmax of 300 mT/m and maximum slew rate of 200 T/m/s). The three participants who completed scans on both systems were females aged 27, 32, and 36 years. Since the groups were matched for age and sex, no additional covariate correction was performed in subsequent statistical analyses.
Reporting on race, ethnicity, or other socially relevant groupings	This study did not include any socially constructed or socially relevant categorization variables. No classifications based on race, ethnicity, or other social groupings were collected or applied in the analysis.
Population characteristics	A total of 39 healthy adults under 40 years of age participated in this study at Massachusetts General Hospital. Participants were divided into two groups: 21 individuals (14 females, 7 males; mean age: 29.0±4.5 years; age range: 19-37) underwent MRI scans using the newly installed 3T Connectome 2.0 MRI scanner (Gmax of 500 mT/m and maximum slew rate of 600 T/m/s). Age- and sex-matched 21 participants (14 females, 7 males; mean age: 28.7±6.2 years; age range: 19-40) were scanned on the 3T Connectome 1.0 scanner (Gmax of 300 mT/m and maximum slew rate of 200 T/m/s).
Recruitment	We recruited young healthy adults between the ages of 19 and 40 years for scans on the Connectome 1.0 and Connectome 2.0 scanners. Participants for the Connectome 1.0 scans were recruited between September 2016 and June 2023. After the Connectome 1.0 scanner was then decommissioned and replaced by the Connectome 2.0 scanner in the same imaging scanner bay, a new group of participants was recruited for scans on the Connectome 2.0 scanner between September 2023 and October 2024. A subset of participants (N=3) were recruited for both scanners within inter-scan intervals of 4-11 months. Our screening process excluded individuals with any history of neurological and psychiatric conditions, encompassing conditions such as dementia, cerebrovascular disease, brain tumors, head injuries, and any other central nervous system disorders. All subjects provided written informed consent prior to participation.
Ethics oversight	The research protocols were reviewed and approved by the Institutional Review Board of Massachusetts General Brigham and were conducted in accordance with the Declaration of Helsinki.

Note that full information on the approval of the study protocol must also be provided in the manuscript.

Field-specific reporting

Please select the one below that is the best fit for your research. If you are not sure, read the appropriate sections before making your selection.

☒ Life sciences ☐ Behavioural & social sciences ☐ Ecological, evolutionary & environmental sciences

For a reference copy of the document with all sections, see nature.com/documents/nr-reporting-summary-flat.pdf

Life sciences study design

All studies must disclose on these points even when the disclosure is negative.

Sample size	Participants were divided into two groups: 21 individuals (14 females, 7 males; mean age: 29.0±4.5 years; age range: 19-37) underwent MRI scans using the newly installed 3T Connectome 2.0 MRI scanner (Gmax of 500 mT/m and maximum slew rate of 600 T/m/s). Age- and sex-matched 21 participants (14 females, 7 males; mean age: 28.7±6.2 years; age range: 19-40) were scanned on the 3T Connectome 1.0 scanner (Gmax of 300 mT/m and maximum slew rate of 200 T/m/s).Three participants completed scans on both systems. Although no formal power

	calculation was performed, the sample size was considered sufficient to compare the two groups and to assess laminar or regional variations in the Connectome 2.0 data.
Data exclusions	No participants were excluded from the analysis. All data acquired were included in the study. There were no specific exclusion criteria, as the study involved healthy adult volunteers who passed screening process.
Replication	The findings are based on original data and were not intended as a replication study. However, consistent scanning and analysis procedures were applied across all participants to ensure reproducibility of the results.
Randomization	Participants were not randomly assigned to scanner groups. Instead, they were scanned on different scanners depending on availability. We matched the groups by age and sex to keep them as similar as possible.
Blinding	Blinding was not applicable to this study. Investigators were aware of group assignments during data analysis, but all image processing steps were performed using same pipelines.

Reporting for specific materials, systems and methods

We require information from authors about some types of materials, experimental systems and methods used in many studies. Here, indicate whether each material, system or method listed is relevant to your study. If you are not sure if a list item applies to your research, read the appropriate section before selecting a response.

Materials & experimental systems

n/a	Involved in the study
☒	☐ Antibodies
☒	☐ Eukaryotic cell lines
☒	☐ Palaeontology and archaeology
☒	☐ Animals and other organisms
☒	☐ Clinical data
☒	☐ Dual use research of concern
☒	☐ Plants

Methods

n/a	Involved in the study
☒	☐ ChIP-seq
☒	☐ Flow cytometry
☐	☒ MRI-based neuroimaging

Plants

Seed stocks	*Report on the source of all seed stocks or other plant material used. If applicable, state the seed stock centre and catalogue number. If plant specimens were collected from the field, describe the collection location, date and sampling procedures.*
Novel plant genotypes	*Describe the methods by which all novel plant genotypes were produced. This includes those generated by transgenic approaches, gene editing, chemical/radiation-based mutagenesis and hybridization. For transgenic lines, describe the transformation method, the number of independent lines analyzed and the generation upon which experiments were performed. For gene-edited lines, describe the editor used, the endogenous sequence targeted for editing, the targeting guide RNA sequence (if applicable) and how the editor was applied.*
Authentication	*Describe any authentication procedures for each seed stock used or novel genotype generated. Describe any experiments used to assess the effect of a mutation and, where applicable, how potential secondary effects (e.g. second site T-DNA insertions, mosiacism, off-target gene editing) were examined.*

Magnetic resonance imaging

Experimental design

Design type	Resting state. No task was performed during the scan.
Design specifications	Each participant underwent a single scan session that included both structural and diffusion MRI acquisitions.
Behavioral performance measures	No behavioral measures were collected.

Acquisition

Imaging type(s)	Structural, Diffusion
Field strength	3T
Sequence & imaging parameters	dMRI data were acquired using a pulsed gradient spin-echo echo-planar-imaging (EPI) sequence. The diffusion times were set to the minimum accessible values for each system for the maximum b-value of 6000 s/mm2, with [illegible] = 13 ms on the Connectome 2.0 scanner and 19 ms on the Connectome 1.0 scanner, respectively, and diffusion-weighted gradient durations (δ) of 6 ms and 8 ms, respectively. The repetition time/echo time (TR/TE) were 3600/53 ms for the Connectome 2.0 scanner and 4000/77 ms for the Connectome 1.0 scanner. The imaging planes for the Connectome 2.0

scanner were axial, whereas those for the Connectome 1.0 scanner were sagittal. Additional common parameters for both scanners included: 2 mm isotropic voxel size, partial Fourier = 6/8, generalized autocalibrating partially parallel acquisition (GRAPPA) acceleration factor = 2, simultaneous multislice (SMS) acceleration factor = 2, anterior-to-posterior phase encoding direction, and adaptive coil combination. To correct for susceptibility-induced distortion, we acquired ten additional non-diffusion-weighted images at the beginning of the dMRI scans with a reversed-phase encoding direction (posterior-to-anterior).

For cortical surface reconstruction and segmentation, high-resolution 3D T1-weighted anatomical images were acquired during the same session. For the Connectome 2.0 scanner, a magnetization-prepared rapid acquisition with gradient echo (MPRAGE) sequence was employed with the imaging parameters: 1 mm isotropic voxel size, TR/ TE = 2500/3.36 ms, TI = 1100 ms, flip angle = 8°, and GRAPPA acceleration factor = 2. For the Connectome 1.0 scanner, we used a multi-echo magnetization-prepared rapid acquisition with gradient echo (MEMPRAGE) sequence with the following parameters: 1 mm isotropic voxel size, TR/TE = 2530/1.15, 3.03, 4.89, and 6.75 ms, TI = 1100 ms, flip angle = 7°, and GRAPPA acceleration factor = 3.

Area of acquisition: Whole brain

Diffusion MRI: ☒ Used ☐ Not used

Parameters: Multi-shell acquisition was performed using eight b-values linearly sampled in gradient strength up to Gmax. For b-values less than 2400 s/mm² (b = 50, 350, 800, and 1500 s/mm²), 32 diffusion encoding directions were acquired. For b-values ≥ 2400 s/mm² (b = 2400, 3450, 4750, and 6000 s/mm²), 64 directions were uniformly distributed on a sphere. Interspersed non-diffusion-weighted images (b = 0 s/mm²) were acquired every 16 diffusion-weighted volumes to enable signal normalization.

Preprocessing

Preprocessing software: dMRI preprocessing was performed using an in-house script based on the DESIGNER pipeline. Tools included MRtrix3 (mrdegibbs) and FSL (topup, eddy). T1-weighted images were processed using FreeSurfer v7.1.4 (recon-all).

Normalization: SANDI metrics were projected to the subject's native cortical surface and aligned to the 'fsaverage' template for group analysis.

Normalization template: FreeSurfer 'fsaverage' surface template was used for inter-subject alignment.

Noise and artifact removal: Gibbs ringing removal (MRtrix3: mrdegibbs), susceptibility and eddy current correction (FSL: topup, eddy), gradient non-linearity correction (in-house MATLAB script) were conducted.

Volume censoring: No volume censoring was performed.

Statistical modeling & inference

Model type and settings: The Kolmogorov-Smirnov test was used to assess data normality. To compare SANDI metrics across scanners, two-sample t-tests were performed on values averaged across the full cortical depth of the entire cortex between the Connectome 2.0 and Connectome 1.0 scanners. To take advantage of the ultra-high-gradient strength of the Connectome 2.0 scanner, all subsequent analyses comparing SANDI metrics to histological atlases and prior cytoarchitectonic and myeloarchitectonic studies were performed exclusively using data from the Connectome 2.0 scanner. Paired t-tests were used to compare intra-soma signal fractions fis and intra-neurite signal fractions fin between the supragranular and infragranular layers. Pearson's correlation analysis was used to assess the correlation between intra-neurite signal fractions fin in the infragranular layer and myelin staining intensity from the myeloarchitecture atlas, using cortical labels defined by Nieuwenhuys' parcellation, excluding labels with missing or very low-intensity data. The infragranular layer was specifically examined because it contains a relatively high density of myelinated axons, which contribute significantly to the intra-neurite signal fin. Additional paired t-tests assessed regional differences in the intra-soma signal fractions fis between the motor and visual cortices across the full cortical depth (0-100%). For each subject, the median intra-soma signal fractions fis value within each region was used due to the relatively small size of the regions of interest. To examine the layer-specific relationship between cortical curvature and intra-soma signal fractions fis, Pearson's correlation analyses were performed separately in the supragranular and infragranular layers, as well as at 10% and 90% cortical depths.

Effect(s) tested: Effects tested included scanner-related differences in intra-soma signal fraction fis and intra-neurite signal fraction fin. For data acquired using the Connectome 2.0 scanner, depth-dependent variations in these metrics were also examined across the cortex.

Specify type of analysis: ☐ Whole brain ☐ ROI-based ☒ Both

Anatomical location(s): Cortical labels included Brodmann areas 4a/4p (motor) and areas 17/18 (visual), defined using FreeSurfer parcellations (aparc.a2009s.annot). In addition, cortical curvature features—sulcal and gyral patterns—were derived from FreeSurfer's 'curv' maps. Cortical labels based on cytoarchitectonic divisions were further defined using Nieuwenhuys' parcellation.

Statistic type for inference (See Eklund et al. 2016): Surface-based cluster-wise analysis was performed on SANDI metrics averaged within cortical labels.

Correction: Multiple comparisons correction was applied using false discovery rate (FDR) correction.

Models & analysis

n/a	Involved in the study
☒	☐ Functional and/or effective connectivity
☒	☐ Graph analysis
☒	☐ Multivariate modeling or predictive analysis
